# Body Composition in Preterm Infants: Current Insights and Emerging Perspectives

**DOI:** 10.3390/children12010053

**Published:** 2025-01-02

**Authors:** Sreekanth Viswanathan, Melissa Thoene, Zaineh Alja’nini, Pradeep Alur, Kera McNelis

**Affiliations:** 1 Division of Neonatology, Department of Pediatrics, Nemours Children’s Hospital, University of Central Florida College of Medicine, Orlando, FL 32827, USA; 2 Division of Neonatology, Department of Pediatrics, University of Nebraska Medical Center, Nebraska Medical Center, Omaha, NE 68198, USA; melissak.thoene@unmc.edu; 3 Division of Neonatology, Department of Pediatrics, MercyKids Children’s Hospital, University of Missouri School of Medicine, Springfield Campus, Springfield, MO 65804, USA; zaineh.aljanini@mercy.net; 4 Division of Neonatology, Department of Pediatrics, Hampden Medical Center, Penn State Health, Enola, PA 17025, USA; palur@pennstatehealth.psu.edu; 5 Division of Neonatology, Department of Pediatrics, Emory University and Children’s Healthcare of Atlanta, Atlanta, GA 30322, USA; kera.michelle.mcnelis@emory.edu

**Keywords:** body composition, preterm infants, postnatal growth, anthropometry

## Abstract

In recent years, significant advancements in respiratory and nutritional care have markedly improved the survival rates of preterm infants and enhanced long-term health outcomes. Despite these improvements, emerging research highlights the lasting impacts of early growth patterns on an individual’s health trajectory. Adults born prematurely face a higher incidence of health issues related to their early birth. The American Academy of Pediatrics recommends that preterm infants should achieve growth rates similar to those of fetuses, with clinicians emphasizing nutrition delivery to help these infants reach their expected weight for gestational age. However, this approach often results in altered body composition, characterized by increased fat mass and decreased fat-free mass compared to full-term infants. Air displacement plethysmography stands out as a highly reliable method for measuring preterm body composition, while DEXA scans, despite their reliability, tend to overestimate body fat. Other methods include bioelectric impedance, isotope dilution, MRI, ultrasound, and skinfold thickness, each with its own strengths and limitations. In this paper, we aim to raise awareness among neonatal clinicians about the importance of achieving acceptable neonatal body composition. We discuss the pros and cons of different body composition measurement methods, the impact of nutrition and other factors on body composition in preterm infants, long-term follow-up data, and the potential use of body composition data to tailor nutritional interventions in NICU and post-discharge settings. This comprehensive approach is designed to optimize health outcomes for preterm newborns by focusing on their body composition from an early stage.

## 1. Introduction

Advancements in respiratory and nutritional care have significantly improved the survival rates of preterm infants, leading to better long-term health outcomes. Despite these improvements, early growth patterns have lasting impacts on an individual’s health. Adults born prematurely often face health issues related to their early birth. The American Academy of Pediatrics advocates for growth rates in preterm infants that mimic those of fetuses, emphasizing growth to achieve expected weight for gestational age [1]. However, this approach can result in altered body composition, characterized by increased fat mass (FM) and decreased fat-free mass (FFM) compared to full-term infants [2].

Body composition in preterm infants is an important factor influencing their immediate and long-term health outcomes. The balance between FFM and FM significantly impacts both neurodevelopmental and metabolic health. Understanding these dynamics is crucial for optimizing care and improving outcomes. Neurodevelopmental outcomes are particularly influenced by the proportion of FFM in preterm infants. Higher levels of FFM are associated with improved neurodevelopment, as evidenced by studies that correlate increased FFM with larger brain size and better cognitive and motor scores at various corrected ages [3,4,5]. In extremely preterm infants, Binder et al. found a significant association between FFM z-scores, including for cerebral biparietal diameter, bone biparietal diameter, and transverse cerebellar diameter at term-equivalent age, indicating enhanced brain growth [3]. Similarly, Ramel et al. demonstrated that early gains in FFM, rather than FM, were linked to improved neurodevelopment at 12 months corrected age in very low birth weight (VLBW) infants [5]. These findings underscore the importance of promoting FFM accrual in preterm infants to support optimal brain development. Metabolic health is another area where body composition plays a crucial role. Preterm infants often exhibit a deficit in FFM and increased adiposity at term-equivalent age compared to full-term infants, potentially predisposing them to metabolic diseases later in life. Specifically, higher FM gains in the early postnatal period are linked to adverse metabolic outcomes such as higher blood pressure at 4 years of age in very preterm infants [6]. Conversely, gains in FFM during the first three months of life are positively associated with better metabolic health, including higher FFM at preschool age, which is considered protective against metabolic syndrome [7]. This emphasizes the need for nutritional strategies that enhance FFM while minimizing excessive fat gain.

Optimizing nutrition is essential to achieving a favorable balance between FFM and FM. Increased protein and energy intake during hospitalization have been associated with higher FFM at discharge, which is a key surrogate marker for organ growth and neurodevelopment [8,9]. However, surplus energy provision can lead to increased adiposity, which is undesirable. Therefore, nutritional management must be carefully tailored to promote healthy growth patterns in preterm infants. Accurate assessment of body composition is crucial for guiding nutritional interventions. Air displacement plethysmography (ADP) provides a reliable method for measuring neonatal body composition, while DEXA scans, though reliable, tend to overestimate body fat. Other methods include bioelectric impedance, isotope dilution, MRI, ultrasound, and anthropometric measurement equations incorporating skinfold thickness. The main objectives of this review are to critically evaluate and summarize the current research on body composition in preterm infants, specifically focusing on the following: (1) the advantages and limitations of various body composition measurement techniques; (2) the influence of postnatal nutrition and other factors on body composition in preterm infants during neonatal intensive care unit (NICU) hospitalization and post-discharge settings, and (3) long-term follow-up data regarding body composition outcomes.

## 2. Methods

This narrative review aims to synthesize the recent evidence linking preterm infants with body composition outcomes. A comprehensive literature search was conducted across four major databases: PubMed, Scopus, Cochrane Central Registry of Controlled Trials, and Google Scholar. The search was restricted to articles published from January 2004 to October 2024. Only studies published in English were included to ensure the accessibility and consistency of the data reviewed. The search strategy employed a combination of specific key terms, including “Preterm infants”, “Postnatal growth”, “body composition”, “anthropometry”, “early nutrition”, and “breastfeeding”. These terms were chosen to capture a broad range of studies focusing on the growth and nutritional aspects influencing body composition in preterm infants.

## 3. Body Composition Measurement Methods

There are several techniques for measuring body composition in preterm infants. Each of these techniques has its own advantages and limitations, and the choice of method may depend on the specific clinical or research context. An overview of the different methods of measuring body composition in preterm infants highlighting their advantages and limitations is given in Table 1.

Air displacement plethysmography (ADP): This method, often using devices like the PEA POD (COSMED, Concord, CA, USA), is a non-invasive, radiation-free method and provides accurate measurements of FM, FFM, and body fat percentage (BF%) in newborns [10,11,12,13,14]. It is suitable for use in clinical settings and research, particularly in preterm infants. The PEA POD system, with its temperature-controlled chamber, enhances comfort and ensures safety for repeated assessments in vulnerable populations such as preterm infants, minimizing their stress. The procedure is quick, typically taking only a few minutes, which is advantageous in clinical settings allowing for rapid assessments with minimal handling. ADP provides comprehensive data by estimating body volume, which is used to calculate body density and subsequently determine FM and FFM.

ADP using PEA POD is considered reliable and has been used to generate reference charts (Norris charts) for body composition assessment in preterm neonates, allowing for the monitoring of growth and nutritional status from 30 weeks GA through the first six months of life [15]. These charts offer percentile curves derived from a large cohort of healthy preterm and term infants, allowing for evaluation of an infant’s body composition against standardized norms for age, sex, and size. This comparison is vital for detecting deviations in growth patterns, potentially indicating nutritional deficits or other health issues. However, ADP does have limitations. Infants who are unstable or require oxygen and intravenous fluids cannot easily undergo PEA POD measurements. It requires specialized equipment, which may not be available in all clinical settings due to cost and space requirements. The equipment also requires regular calibration and maintenance to ensure measurement accuracy, which can be resource intensive.

Dual-energy X-ray absorptiometry (DXA): DXA is considered a gold standard for body composition assessment, providing detailed information on bone mineral density, FM, and lean body mass. However, it involves exposure to low levels of radiation, which may limit its use in preterm infants [16,17,18,19]. Movement artifacts due to the infants’ propensity to move during scans can compromise the accuracy and precision of measurements, although advanced analysis techniques have been developed to address these issues [18]. Additionally, DXA is known to overestimate fat content, showing poor agreement with methods like ADP and isotope dilution, leading to inconsistent results [16,17,19]. The accuracy of DXA is also affected by technical and operator variability, with significant differences observed due to varying skills and software used for analysis [20]. Moreover, DXA’s validation for use in preterm infants is limited, and its results may not be directly comparable to other validated methods [17,21]. Finally, the high cost and limited availability of DXA machines in clinical settings further restrict their routine use for body composition assessment in the preterm population [22].

Bioelectrical impedance analysis (BIA): BIA is a valuable tool for assessing body composition in preterm infants by measuring resistance and reactance to estimate total body water (TBW) and FFM [23,24,25]. It is non-invasive, portable, cost-effective, and particularly useful in NICUs for monitoring fluid and nutritional status [23,24,25]. BIA has been validated against reference methods such as deuterium and bromide dilution, showing good correlation with TBW and extracellular water but with notable biases and wide limits of agreement [23,26]. However, its accuracy is influenced by several factors, including the need for specific resistivity coefficients, electrode positioning, and the dynamic nature of body composition in preterm infants [23,24,27]. The lack of standardized protocols for electrode placement and hydration status further complicates its reliability. Validation studies comparing BIA to ADP (PEA POD) demonstrate improved prediction accuracy in older infants but not significantly in those younger than three months [23,26]. This limitation is likely related to the dynamic changes in body water content in early life. Though BIA provides practical advantages for body composition assessment in preterm infants, its clinical utility is limited by methodological challenges, necessitating further validation and standardization for broader applications.

Isotope dilution techniques: Isotope dilution techniques are a validated and reliable method for assessing body composition in preterm infants. This method involves administering a known quantity of a stable isotope, such as deuterium oxide (D2O) or oxygen-18 (18O), and measuring its dilution in body fluids like saliva or urine to estimate TBW. The TBW measurement is then used to calculate FFM and FM using established hydration constants. Isotope dilution is considered the gold standard for measuring TBW, having been validated against other methods such as ADP and DXA [17,28,29]. The technique is non-invasive and safe, making it suitable for medically fragile populations, and it avoids ionizing radiation, allowing for repeated measurements [13,17]. However, it requires specialized equipment and expertise, which can be costly and time-consuming. Additionally, the accuracy of the method can be influenced by the infant’s hydration status, necessitating careful control [10,13,17]. While isotope dilution techniques offer significant advantages in terms of safety and precision and are often used in research, they are less practical for routine clinical use due to their complexity and cost.

Magnetic resonance imaging (MRI): MRI is a sophisticated tool for assessing body composition in preterm infants, offering detailed images of regional fat distribution and organ size without the use of ionizing radiation, making it safe for repeated use [10,30,31,32,33]. MRI’s high-resolution capabilities allow for precise quantification of various tissue types, such as lean body mass, adipose tissue, and brown adipose tissue, and enable the assessment of regional body composition to better understand growth patterns and nutritional needs [10,30,31,32,33]. Techniques such as the 2-Point Dixon and free-breathing radial sequences are employed to create fat and water images, which are then analyzed to quantify tissue types. These methods have been validated in studies demonstrating high correlation with body weight and good repeatability for adiposity measurements [10,30,31]. However, MRI is limited by its high cost and the need for specialized equipment and personnel. The requirement for infants to remain still during scans can be challenging, often necessitating sedation or other protocols to reduce motion artifacts. MRI is a feasible, accurate, and repeatable method for assessing body composition in preterm infants, with significant advantages in detail and safety, but it is limited by practical and logistical challenges [10,13,31,32].

Ultrasound: Ultrasound techniques are increasingly utilized for assessing body composition in preterm infants due to their non-invasive nature and practicality in clinical settings. This method employs high-frequency sound waves to capture detailed images of soft tissue structures, focusing on measuring subcutaneous fat and muscle thickness at various anatomical sites such as the biceps, triceps, subscapular, and abdominal regions. These measurements can be performed at bedside, making them particularly suitable for fragile preterm infants in NICUs [34,35]. Studies have validated the reliability and reproducibility of ultrasound in measuring subcutaneous fat and muscle thickness. For example, Buck et al. demonstrated high inter-rater and intra-rater reliability, with intraclass correlation coefficients ranging from 85.8 to 99.9 for subcutaneous fat measurements in very preterm infants [34]. Additionally, McLeod et al. found ultrasound effective in detecting influences of macronutrient intake on tissue accretion rates, highlighting its clinical utility [35]. Advantages of ultrasound include its non-invasive, portable nature, absence of ionizing radiation, and cost-effectiveness compared to other imaging modalities like MRI and DXA [22,34,35]. However, limitations include the need for trained personnel and standardized protocols, as variability in measurement techniques can impact the consistency of results. Moreover, ultrasound may not provide comprehensive whole-body composition data as accurately as other methods [22,36].

Anthropometric measurement equations: Estimating body composition in preterm infants using anthropometric measurements is an important aspect of neonatal care, yet its reliability varies across different methods. Routine anthropometric measures, such as weight and length indices, are traditionally employed but often fail to accurately reflect body composition in preterm infants due to their unique growth patterns. Ramel et al. highlighted that while body weight without length adjustment strongly correlates with FFM, weight/length indices do not reliably indicate %BF in preterm infants near birth (R^2^ = 0.97 for FFM) [37]. Similarly, studies like the INTERGROWTH-21st project have found that simple anthropometric ratios are inadequate for predicting neonatal body composition, necessitating more sophisticated techniques [38]. Upper arm anthropometry, including mid-upper arm circumference (MUAC), has also been assessed but with inconsistent results. Pereira-da-Silva et al. demonstrated that upper arm measures are not valid predictors of regional body composition, showing poor correlation with MRI-measured cross-sectional arm areas (r^2^ < 0.56) [39]. Conversely, mid-arm circumference (MAC) has been identified as a more reliable and cost-effective measure for monitoring body fat deposition, accounting for 60.4% of the variability in %BF by ADP [40]. A systematic review by Yumani et al. concluded that conventional anthropometric measures, including weight and length indices, body circumferences, and skinfold thickness, are inadequate for representing body composition in preterm infants [17]. They suggested that advanced methods like ADP, DXA, and isotope dilution provide more trustworthy assessments, though results may not always be comparable [17]. While mid-arm circumference offers a practical approach for estimating body composition in preterm infants, other routine anthropometric measurements are less reliable. The integration of simple anthropometric methods with advanced techniques could enhance the accuracy and reliability of body composition assessments in preterm infants.

## 4. Impact of Postnatal Nutrition on Body Composition

Given the strong association between FFM and neurodevelopmental outcomes in preterm infants, [3,4,5,6] the question arises: how can FFM growth be increased in this population? Nutrition provision is a modifiable factor, and numerous studies have explored the relationship between nutrition and body composition in preterm infants. Research has primarily focused on several key areas: parenteral nutrition (PN), enteral feeding—including the use of human milk and its fortification—and post-discharge nutritional care.

PN is frequently initiated immediately for the smallest and youngest preterm infants and is continued until they achieve enteral autonomy [41]. However, the duration of PN has been shown to negatively correlate with FFM z-scores at term corrected age, as indicated by a case-control study [42]. Liotto et al. examined the transition from parenteral to enteral nutrition, finding that VLBW infants with higher weight growth velocity during this transition received greater parenteral protein intake and subsequently had a higher percentage of FFM at term corrected age [43]. The Nutritional Evaluation and Optimization in Neonates (NEON) trial explored the effects of amino acid provision and found no significant difference in FFM between infants receiving higher versus lower amino acid doses [44]. However, there was a significant interaction effect with lipid emulsion. The investigators concluded that immediate high-dose amino acid provision offers no benefit and may pose potential harm. This suggests that while immediate high doses of amino acids are not advantageous, maintaining higher protein provision during the transition from PN to enteral feeding could enhance FFM outcomes.

Additionally, a secondary analysis from the NEON trial revealed that infants predominantly fed formula had higher non-adipose tissue mass at term age compared to those exclusively fed human milk, even after adjusting for total nutritional intake [45]. The authors have explained that unfortified human milk has a lower protein:energy ratio than preterm formula and this may explain the lower non-adipose tissue in the breastfed compared with formula-fed infants [45]. Conversely, a study of 379 NICU infants assessing growth, body composition via ADP, and neurodevelopmental outcomes found that human milk feeding at discharge was significantly associated with higher FFM z-scores [46]. A pilot study also suggested that a higher proportion of donor to parental human milk intake was negatively associated with BF% in VLBW infants [47]. These findings emphasize the importance of tailored nutritional strategies in supporting the growth and body composition of preterm infants.

Research indicates that protein intake and its ratio with carbohydrate and energy provision play crucial roles in the growth and body composition of preterm infants. In a study involving 50 preterm infants born before 32 weeks gestational age, it was found that the protein to carbohydrate ratio was a significant predictor of FFM at 34–37 weeks corrected age, even after adjusting for factors such as birth weight, birth weight z-score, and postmenstrual age [48]. Similarly, Ramel et al. demonstrated that increased energy and protein intake during the first week after birth, as well as sustained protein intake throughout NICU hospitalization, were linked to higher FFM z-scores at discharge in a cohort of 103 VLBW infants [8]. Additionally, a study of 141 preterm infants born before 35 weeks gestational age showed that a higher protein to energy ratio at days 10 and 21 was significantly associated with a reduced risk of FFM deficit [49]. These findings underscore the importance of optimizing early and sustained protein and energy intake in the nutritional management of preterm infants to promote healthy growth and development. According to the American Society for Parenteral and Enteral Nutrition, a protein to energy ratio of 3.0 to 3.5 g/100 kcal is recommended for preterm infants to support adequate growth and body composition [50].

One limitation of many studies examining the nutritional effects of human milk fortification is the lack of direct human milk analysis, with many relying on assumed macronutrient values based on published averages. This can lead to inaccuracies because human milk is a complex and variable biological system, and its composition can fluctuate significantly [51]. Although human milk is the preferred source of nutrition for preterm infants, treating it as a homogenous nutritional product in studies may overlook the complex interplay of various components such as microbiota, xenobiotics, hormones, and immune factors [52]. For instance, a study involving 82 very preterm infants found no difference in body composition at term corrected age when analyzing their exposure to different concentrations of human milk oligosaccharides, suggesting that these complex interactions may influence study outcomes [53].

Fortification is added to human milk to provide additional nutrients, such as protein, calcium, phosphorus, and sodium, to create a more complete nutritional source for preterm infants that better mimics intrauterine provision [41]. There is significant inter-center variability in feeding protocols. The “IMPACT” trial (Increased Milk Protein to Accrue Critical Tissue) by Salas et al. randomized extremely preterm infants to receive either an unfortified human milk diet or a human milk-based fortification starting on the second day of life [54]. At the study’s endpoint, the FFM z-score was higher in the intervention group, although the difference did not reach statistical significance. Other anthropometric markers of growth were improved in the intervention group. It is possible that if all study subjects had been able to undergo body composition measurements—since the use of ADP was limited by respiratory contraindications—the difference in FFM might have achieved statistical significance [55]. Other studies have conducted direct human milk analysis to evaluate different milk fortification strategies and their impact on body composition in preterm infants. A small study found that VLBW infants receiving targeted individualized human milk fortification, as opposed to standard fortification, had higher FFM and flank skin fold thickness at 37 weeks corrected age or prior to discharge [56]. A randomized controlled trial involving 40 infants compared standard and targeted human milk fortification strategies using bovine multi-component milk and modular nutrient supplements, found no statistically significant difference in BF% or weight between the groups [57]. Similarly, another study involving 60 preterm infants born before 32 weeks gestational age, randomized to standard or individualized fortification, did not find a difference in body composition at 40 weeks or at four months corrected age [58]. Additionally, Piemontese et al. evaluated the impact of human milk on body composition, taking into account the variability in macronutrient content [59]. In this study, 73 VLBW infants received either parental or donor milk that was target fortified with bovine multicomponent and modular nutrient fortifiers after analysis. It was found that being fed human milk constituting 50% or more of total milk intake was associated with an increase in FFM percentage at term corrected age [59].

Multiple studies have examined the body composition of former preterm infants in the first year of life following NICU discharge [60,61]. Some of these studies have compared the use of preterm formula versus term formula as post-discharge nutrition [62,63]. For instance, the 2012 study by Roggero et al. found that appropriate-for-gestational-age former preterm infants who were randomized to receive preterm (nutrient-enriched) formula after NICU discharge exhibited increased FFM. However, it is important to note that some of these older studies may not fully reflect current growth patterns, as there have been concurrent changes in NICU care practices over the years [64,65].

## 5. Sex Differences in Body Composition

Sexual dimorphism in body composition is a well-documented phenomenon in adults, with distinct patterns of fat distribution linked to health outcomes such as diabetes [66]. In adults, women typically have more subcutaneous fat, while men have more visceral fat, which is associated with higher diabetes risk [66]. Hence, paying attention to body composition in both sexes has health implications. Let us examine whether similar sex differences exist in the body composition of term and preterm infants and how current nutritional practices might influence these differences.

Intrauterine body composition studies using MRI have revealed significant sexual dimorphism between 30–36 weeks of gestation. Males were found to have higher body mass, FFM, and FFM%, while females showed a higher FM% [67]. These findings underscore the importance of considering sex-specific growth trajectories during prenatal assessments. The Multicenter Infant Body Composition Reference Study (MIBCRS) further supports these findings in infants from birth to 24 months, noting higher FM indices and lower FFM indices in female infants [68]. This study reinforced the intrauterine body composition results of sexual dimorphism in term infants. The body composition of preterm infants is also specific to their sex. The INTERGROWTH-21st study reported higher FM% in female preterm infants compared to males, with males having more FFM [38]. Female infants had more significant skin fold thickness measurements and larger central to total skinfold ratio (CTS) values in every gestational age group, with significant differences from 35 weeks gestation onwards. The CTS ratio is used to index the central pattern of adiposity distribution, especially in the thoracic and abdominal subcutaneous fat [69]. These studies highlight that even preterm infants exhibit adult-like patterns of fat distribution at birth.

Preterm infants’ body composition at corrected term gestation differs significantly from term infants, often showing higher total body fat and less FFM indicating altered growth patterns [2]. MRI studies have shown that extremely preterm infants have less subcutaneous adipose tissue and increased intra-abdominal fat, which is linked to insulin resistance [70]. Nutrition plays a crucial role in the development of body composition in preterm infants. A prospective cohort study found that preterm infants fed their mother’s own milk (MOM) had an FM% similar to term infants, whereas those fed formula had a higher FM% [71]. This suggests that sex-specific nutrition might be beneficial, as MOM is naturally tailored to the infant’s sex, unlike formula. Further studies confirm that sex-specific differences in body composition, such as higher FM% in female term infants, are not observed in preterm infants fed formula [72]. This lack of differentiation suggests that unisex nutritional approaches may not adequately support the distinct growth needs of preterm infants [73]. The reasons for these sex differences are not fully understood but may involve hormonal factors like fetal testosterone production.

## 6. Impact of Body Composition on Long-Term Clinical Outcomes

While the promotion of adequate growth across all anthropometric measurements is a primary goal of neonatal nutrition support, ensuring appropriate body composition is equally important. Preterm infants are likely to undergo significant physiological changes during NICU hospitalization, affecting body composition, including the proportions of FM and FFM. Given that various nutritional and non-nutritional clinical interventions can influence growth, clinicians must carefully consider the implications of these interventions on the resulting body composition.

The relationship between neonatal body composition and longitudinal outcomes continues to be a focus of research. Increased accrual of FFM, which includes lean body mass, is suggested as a crude surrogate indicator of proper organ and tissue development in the neonatal period. For example, in extremely low birth weight (ELBW) infants, increased FFM but lower FM z-scores for age, as measured by ADP, have been associated with larger physical brain size [3]. Increasing FFM during NICU hospitalization for VLBW infants has been linked to improved cognitive and motor scores on the Bayley Scales of Infant Development at one-year corrected age, even after adjusting for birth size, age, and clinical factors [5,8]. Similarly, in a cohort of infants born before 32 weeks gestation or with VLBW, higher increases in FM, FFM, and BF% from 32 to 37 weeks gestational age were associated with lower odds of developing retinopathy of prematurity at stages greater than two [74]. Notably, while relationships between increased FM gain and neurocognitive benefits were not observed [5,8], suggesting that increasing adipose tissue beyond normal proportions relative to FFM accrual during the neonatal period does not confer neurocognitive advantages, data from a cohort of infants born before 35 weeks gestational age indicated that gains in BF% from term to 3–4 months corrected age were associated with lower memory performance at 4 years of age [75]. These findings underscore the significance of body composition and highlight the time sensitivity of such changes in early life, emphasizing the potential long-term cognitive implications of altered adiposity in preterm infants.

Current knowledge regarding early neonatal nutrition and body composition suggests that these factors also contribute to programming for body composition and function in later life. For example, a study of infants born before 32 weeks gestation or with VLBW identified a relationship between FFM within the first 3 months of corrected age and FFM at 4–7 years of age [7]. Additionally, data have shown lower lean body mass and height in adults who were born with VLBW [76]. However, increased early neonatal protein provision in VLBW infants has been associated with greater adult FFM and a higher resulting metabolic rate [77].

More data are being collected to understand the distribution of adipose tissue in preterm versus term-born infants and the relationship between overall neonatal body composition and later cardiometabolic health. For instance, among infants born before 32 weeks gestation or with VLBW, higher FM gain from term to 4 months corrected age has been positively associated with blood pressure at 4 years of age [6]. Browning of white adipose tissue and adaptation to respond to oxidative stress is known to play a role in critical illness in adults, but this physiology or potential mechanism is not yet described in critically ill preterm infants [78]. Future studies will not only examine birth weight or weight gain trajectories during NICU hospitalization but also body composition and its later effects on glucose metabolism, blood lipid concentrations, and cardiovascular function.

## 7. Body Composition Data for Optimizing Nutrition for Preterm Infants

The nutritional management of VLBW and preterm infants is a critical component of care during NICU hospitalization and continues to be important post-discharge. The use of body composition data, specifically measurements of FFM and FM, has become increasingly valuable in guiding nutritional interventions that enhance individualized care and improve long-term outcomes for these infants.

In the NICU, traditional measures such as weight and length do not differentiate between FFM and FM. Body composition measurements provide a more accurate assessment of FFM and FM, which can help to optimize nutritional strategies. For instance, increased energy and protein intake during hospitalization are associated with higher FFM at discharge, which is vital for organ growth and neurodevelopment in preterm infants. Monitoring body composition allows for adjustment of macronutrient intake, ensuring that infants receive adequate protein and energy to support FFM accretion without excessive fat gain [8,48]. The frequency of body composition monitoring is vital for timely nutritional adjustments aimed at promoting FFM accretion while minimizing excessive fat gain. Studies suggest that monitoring every 2–4 weeks is effective in detecting significant changes in body composition, allowing for timely adjustments in nutritional interventions [79]. Frequent monitoring supports clinicians in adjusting macronutrient intake, particularly protein and energy, to support optimal growth and development [80].

Post-NICU discharge, individualized nutritional strategies can continue to be informed by body composition data. Feeding preterm infants with protein- and mineral-enriched post-discharge formulas has been shown to improve lean mass and bone mineral content compared to standard term formulas [81]. Furthermore, body composition data can help identify infants at risk of postnatal growth faltering and guide the use of fortified human milk or specialized formulas to support enhanced growth and neurodevelopment [82].

While protein-enriched formulas are effective in promoting lean mass accretion and improving growth parameters [83], they can also lead to gastrointestinal disturbances such as increased frequency of spit-ups and softer stool consistency [84,85]. Additionally, there is a potential risk of metabolic burden associated with protein overprovision, which can lead to long-term issues such as insulin resistance and obesity [86,87]. The use of high-quality protein formulas, which include components like alpha-lactalbumin and partially hydrolyzed whey, can mitigate some of these risks by improving protein quality and reducing the overall protein intake required [84].

Incorporating body composition data into the nutritional management of preterm infants enables precise and individualized care, improving short-term growth and long-term health outcomes by tailoring interventions based on body composition. As research advances, this approach will likely become a standard component of neonatal care, promoting optimal growth, neurodevelopment, and minimizing adverse metabolic risks.

## 8. Routine Clinical Use of Body Composition Strategies for Individualized Care in NICU

The integration of body composition strategies into NICUs for preterm infants is an emerging focus aimed at enhancing individualized care and precision nutrition. To address this, advanced body composition assessment methods, including ADP, bioelectrical impedance analysis, and skinfold measurements, have been explored for their clinical utility in preterm infants. We have described the implementation process for routine body composition using ADP into routine NICU clinical practice previously [88]. These techniques provide more precise insights into the nutritional status and growth patterns important for optimizing neurodevelopmental and metabolic outcomes [88,89,90,91]. Despite the potential benefits, routine clinical adoption of these methods remains limited due to challenges such as the need for standardized protocols and additional workload [79,88,91]. Though body composition strategies offer substantial potential for advancing individualized care and precision nutrition in the NICU, further research and the establishment of standardized protocols are crucial for their complete integration into routine clinical practice. By overcoming these challenges, healthcare providers can significantly improve the quality of care for preterm infants, promoting healthier growth trajectories and better long-term health outcomes.

## 9. Future Directions and Addressing Knowledge Gaps in Body Composition of Preterm Infants

Understanding body composition in preterm infants is vital due to its significant impact on both immediate and long-term health outcomes. To effectively integrate body composition assessment into routine care and improve health outcomes, several key research areas and clinical practices must be addressed.

A major challenge is the lack of standardized protocols for body composition assessment across clinical settings. Techniques such as ADP offer valuable insights but require consistent application and interpretation standards. While increased energy and protein intake is known to enhance FFM, optimal nutritional strategies for achieving favorable body composition remain unclear. Research should focus on the effects of different macronutrient quantities and ratios as well as the timing of nutritional interventions on body composition, including human milk fortification and specialized formulas post-discharge. This will aid in developing personalized nutrition plans that support healthy growth trajectories.

Another critical area is understanding the link between neonatal body composition and long-term health outcomes like neurodevelopment and metabolic health. Studies should aim to determine how early body composition affects cognitive and motor development and the risk of metabolic disorders later in life. Longitudinal studies tracking body composition from the neonatal period through adulthood will provide essential insights into these relationships.

For routine adoption of body composition strategies in NICUs, challenges such as equipment availability, staff training, and integration of data into electronic health records must be addressed. Developing cost-effective and user-friendly technologies for body composition assessment will facilitate their widespread use. Additionally, educational programs for clinicians on data interpretation and application will enhance clinical utility.

Beyond nutrition, factors such as medical interventions, maternal health, and genetic predispositions may influence body composition in preterm infants. Further research is needed to understand these influences and their interaction with nutritional strategies on growth and development. Future studies should also consider the moderating effects of concurrent medical interventions and baseline demographic variables and exposures, such as maternal hypertension or diabetes, and intrauterine growth restriction, which impact outcomes. Sex differences in body composition from the intrauterine period through infancy influence nutritional strategies for preterm infants. Current unisex nutritional practices may not be optimal, highlighting the need for sex-specific nutritional requirements to support healthy growth and development. Research limitations include issues such as attrition, feasibility or contraindications to body composition measurement in clinical care, and standardization of results to reference trajectories accounting for gestational age and sex differences. Addressing these areas will enhance our understanding of body composition’s role in the health and development of preterm infants, leading to more effective interventions and improved long-term health outcomes.

## 10. Conclusions

Advancing body composition strategies in the care of preterm infants promises significant improvements in individualized care and precision nutrition. Addressing knowledge gaps through targeted research and standardized protocol development will empower healthcare providers to enhance care quality. Integrating body composition assessments into routine practice will better support the growth and development of preterm infants, ultimately leading to healthier outcomes throughout their lives. As the field advances, these strategies are likely to become integral to neonatal care, promoting optimal growth and minimizing long-term health risks.

## Figures and Tables

**Table 1:** An overview of the different methods of measuring body composition in preterm infants highlighting their advantages and limitations.

Measurement Technique	Advantages	Limitations
Air displacement plethysmography (ADP)	- Non-invasive and quick - No radiation exposure, suitable for repeated measurements - Provides data on FM and FFM - Availability of standardized normative body composition growth charts (Norris charts)	- Requires specialized equipment - Requires regular calibration and maintenance - May be less accurate in very small or unstable infants - Not practical for infants receiving oxygen or IV fluids
Dual-energy X-ray absorptiometry (DXA)	- High precision and accuracy - Provides detailed information on bone mineral content, FM, and lean mass	- Exposure to low levels of radiation - Requires infants to remain still - DXA may overestimate fat content - DXA accuracy varies with operator skill - Limited availability and high cost - Limited validation in preterm infants
Bioelectrical impedance analysis (BIA)	- Portable and relatively inexpensive - Quick and easy to perform	- Accuracy can be affected by hydration status and evolving nature of body composition in preterm infants - Electrode placement influences accuracy - Less precise in very preterm infants, compared to reference standards, need more validation and standardization
Isotope dilution techniques (total body water (TBW) analysis)	- Safe, non-invasive, and radiation-free - Precise estimate of TBW, and FFM	- Need for specialized equipment and expertise to administer the isotope and analyze the samples - Time-consuming and expensive - Accuracy can be affected by hydration status
Magnetic resonance imaging (MRI)	- Safe, non-invasive, and radiation-free - Highly detailed images, high accuracy - Can assess organ structures and regional adiposity (subcutaneous vs. visceral), and can track changes over time	- Expensive and not widely available - Requires sedation or infant stillness - Lengthy procedure time
Ultrasound	- Safe, non-invasive, and radiation-free - Portable and relatively inexpensive	- Need for trained personnel, operator-dependent measurement variability - Lack of standardized protocols - Limited ability to assess deep body fat or comprehensive whole-body composition data
Anthropometric measurements equations	- Easy to obtain, practical for generalized use - Cost effective	- Inconsistent and less reliable - Inadequate representation of whole body composition

Abbreviations: FM: fat mass, FFM: fat-free mass.

## Data Availability

Data are contained within the article.

## References

[1] American Academy of Pediatrics Committee on Nutrition (2004). Pediatric Nutrition Handbook.

[2] Johnson M.J., Wootton S.A., Leaf A.A., Jackson A.A. (2012). Preterm birth and body composition at term equivalent age: A systematic review and meta-analysis. Pediatrics.

[3] Binder C., Buchmayer J., Thajer A., Giordano V., Schmidbauer V., Harreiter K., Klebermass-Schrehof K., Berger A., Goeral K. (2021). Association Between Fat-Free Mass and Brain Size in Extremely Preterm Infants. Nutrients.

[4] Anne F.C., Laure C., Cyril F., Matthieu H., Dominique D., Jean C.R. (2018). Deficit of Fat Free Mass in Very Preterm Infants at Discharge is Associated with Neurological Impairment at Age 2 Years. J Pediatr..

[5] Ramel S.E., Gray H.L., Christiansen E., Boys C., Georgieff M.K., Demerath E.W. (2016). Greater Early Gains in Fat-Free Mass, but Not Fat Mass, Are Associated with Improved Neurodevelopment at 1 Year Corrected Age for Prematurity in Very Low Birth Weight Preterm Infants. J. Pediatr..

[6] Pfister K.M., Zhang L., Miller N.C., Ingolfsland E.C., Demerath E.W., Ramel W.E. (2018). Early body composition changes are associated with neurodevelopmental and metabolic outcomes at 4 years of age in very preterm infants. Pediatr. Res..

[7] Nehab S.R.G., Villela L.D., Abranches A.D., Junior S.C.S., Soares F.V.M., Moreira M.E.L. (2024). Association Between Preterm Infant Body Composition in the First 3 months of Life and Preschool Age: A Cohort Study. Eur. J. Pediatr..

[8] Ramel S.E., Haapala J., Super J., Boys C., Demerath E.W. (2020). Nutrition, Illness and Body Composition in Very Low Birth Weight Preterm Infants: Implications for Nutritional Management and Neurocognitive Outcomes. Nutrients.

[9] Brinkis R., Albertsson-Wikland K., Šmigelskas K., Vanckavičienė A., Aldakauskienė I., Tamelienė R., Verkauskienė R. (2024). Impact of Nutrient Intake on Body Composition in Very Low-Birth Weight Infants Following Early Progressive Enteral Feeding. Nutrients.

[10] Andrews E.T., Beattie R.M., Johnson M.J. (2019). Measuring Body Composition in the Preterm Infant: Evidence Base and Practicalities. Clin. Nutr..

[11] Ellis K.J., Yao M., Shypailo R.J., Wong W.W., Heird W.C. (2023). Body-composition assessment in infancy: Air-displacement plethysmography compared with a reference 4-compartment model. Am. J. Clin. Nutr..

[12] Fields D.A., Gilchrist J.M., Catalano P.M., Giannì M.L., Roggero P.M., Mosca F. (2022). Air-displacement plethysmography: Here to stay. Curr. Opin. Clin. Nutr. Metab. Care.

[13] Nagel E., Hickey M., Teigen L., Kuchnia A., Curran K., Soumekh L., Earthman C., Demerath E., Ramel S. (2020). Clinical Application of Body Composition Methods in Premature Infants. JPEN J. Parenter. Enteral Nutr..

[14] Jerome M.L., Valcarce V., Lach L., Itriago E., Salas A.A. (2023). Infant Body Composition: A Comprehensive Overview of Assessment Techniques, Nutrition Factors, and Health Outcomes. Nutr. Clin. Pract..

[15] Norris T., Ramel S.E., Catalano P., Caoimh C.N., Roggero P., Murray D., Fields D.A., Demerath E.W., Johnson W. (2019). New Charts for the Assessment of Body Composition, According to Air-Displacement Plethysmography, at Birth and Across the First 6 Mo of Life. Am. J. Clin. Nutr..

[16] Yumani D.F.J., de Jongh D., Lafeber H.N., van Weissenbruch M.M. (2021). A Comparative Study Using Dual-Energy X-Ray Absorptiometry, Air Displacement Plethysmography, and Skinfolds to Assess Fat Mass in Preterms at Term Equivalent Age. Eur. J. Pediatr..

[17] Yumani D.F.J., de Jongh D., Ket J.C.F., Lafeber H.N., van Weissenbruch M.M. (2023). Body Composition in Preterm Infants: A Systematic Review on Measurement Methods. Pediatr. Res..

[18] Shepherd J.A., Sommer M.J., Fan B., Powers C., Stranix-Chibanda L., Zadzilka A., Basar M., George K., Mukwasi-Kahari S., Siberry G. (2017). Advanced Analysis Techniques Improve Infant Bone and Body Composition Measures by Dual-Energy X-Ray Absorptiometry. J. Pediatr..

[19] Picaud J.C., Rigo J., Nyamugabo K., Milet J., Senterre J. (1996). Evaluation of Dual-Energy X-Ray Absorptiometry for Body-Composition Assessment in Piglets and Term Human Neonates. Am. J. Clin. Nutr..

[20] Koo W.W., Walters J., Bush A.J. (1995). Technical Considerations of Dual-Energy X-Ray Absorptiometry-Based Bone Mineral Measurements for Pediatric Studies. J. Bone Miner. Res..

[21] van Beijsterveldt I.A.L.P., Beunders V.A.A., Bijlsma A., Vermeulen M.J., Joosten K.F.M., Hokken-Koelega A.C.S. (2022). Body Composition Assessment by Air-Displacement Plethysmography Compared to Dual-Energy X-Ray Absorptiometry in Full-Term and Preterm Aged Three to Five Years. J. Clin. Med..

[22] Sheean P., Gonzalez M.C., Prado C.M., McKeever L., Hall A.M., Braunschweig C.A. (2020). American Society for Parenteral and Enteral Nutrition Clinical Guidelines: The Validity of Body Composition Assessment in Clinical Populations. JPEN J. Parenter. Enteral Nutr..

[23] Collins C.T., Reid J., Makrides M., Lingwood B.E., McPhee A.J., Morris S.A., Gibson R.A., Ward L.C. (2013). Prediction of body water compartments in preterm infants by bioelectrical impedance spectroscopy. Eur. J. Clin. Nutr..

[24] Toledo L.F.M., Medeiros T.R., Vieira A.A., Coca Velarde L.G. (2022). Evaluation of the bioimpedance technique in newborns with a focus on electrode positioning: A prospective, randomized, crossover study. Nutr. Clin. Pract..

[25] Pinheiro-Castro N., Ramos-Silva T., de Carvalho Rondó P.H., Ward L.C. (2024). Determination of resistance at zero and infinite frequencies in bioimpedance spectroscopy for assessment of body composition in babies. Physiol. Meas..

[26] Lingwood B.E., Storm van Leeuwen A.M., Carberry A.E., Fitzgerald E.C., Callaway L.K., Colditz P.B., Ward L.C. (2012). Prediction of fat-free mass and percentage of body fat in neonates using bioelectrical impedance analysis and anthropometric measures: Validation against the PEA POD. Br. J. Nutr..

[27] Lyons-Reid J., Ward L.C., Kenealy T., Cutfield W. (2020). Bioelectrical impedance analysis-an easy tool for quantifying body composition in infancy?. Nutrients.

[28] Bandara T., Hettiarachchi M., Liyanage C., Amarasena S., Wong W.W. (2015). Body Composition Among Sri Lankan Infants by ^18^O Dilution Method and the Validity of Anthropometric Equations to Predict Body Fat Against ^18^O Dilution. BMC Pediatr..

[29] Wells J.C.K., Davies P.S.W., Fewtrell M.S., Cole T.J. (2020). Body Composition Reference Charts for UK Infants and Children Aged 6 Weeks to 5 Years Based on Measurement of Total Body Water by Isotope Dilution. Eur. J. Clin. Nutr..

[30] Dyke J.P., Garfinkel A.C., Groves A.M., Kovanlikaya A. (2018). High-Resolution Rapid Neonatal Whole-Body Composition Using 3.0 Tesla Chemical Shift Magnetic Resonance Imaging. Pediatr. Res..

[31] Armstrong T., Ly K.V., Ghahremani S., Calkins K.L., Wu H.H. (2019). Free-Breathing 3-D Quantification of Infant Body Composition and Hepatic Fat Using a Stack-of-Radial Magnetic Resonance Imaging Technique. Pediatr. Radiol..

[32] Bauer J.S., Noël P.B., Vollhardt C., Much D., Degirmenci S., Brunner S., Rummeny E.J., Hauner H. (2015). Accuracy and Reproducibility of Adipose Tissue Measurements in Young Infants by Whole Body Magnetic Resonance Imaging. PLoS ONE.

[33] Flanagan E.W., Altazan A.D., Carmichael O.T., Hu H.H., Redman L.M. (2021). Practical Application of in Vivo MRI-based Brown Adipose Tissue Measurements in Infants. Obesity.

[34] Buck C.O., Santoro K.L., Shabanova V., Martin C.R., Taylor S.N. (2024). Establishing Feasibility and Reliability of Subcutaneous Fat Measurements by Ultrasound in Very Preterm Infants. Pediatr. Res..

[35] McLeod G., Geddes D., Nathan E., Sherriff J., Sherriff K., Hartmann P. (2013). Feasibility of Using Ultrasound to Measure Preterm Body Composition and to Assess Macronutrient Influences on Tissue Accretion Rates. Early Hum. Dev..

[36] Nagel E., Hickey M., Teigen L., Kuchnia A., Holm T., Earthman C., Demerath E., Ramel S. (2021). Can Ultrasound Measures of Muscle and Adipose Tissue Thickness Predict Body Composition of Premature Infants in the Neonatal Intensive Care Unit?. JPEN J. Parenter. Enteral Nutr..

[37] Ramel S.E., Zhang L., Misra S., Anderson C.G., Demerath E.W. (2017). Do Anthropometric Measures Accurately Reflect Body Composition in Preterm Infants?. Pediatr. Obes..

[38] Villar J., Puglia F.A., Fenton T.R., Ismail L.C., Staines-Urias E., Giuliani F., Ohuma E.O., Victora C.G., Sullivan P., Barros F.C. (2017). Body composition at birth and its relationship with neonatal anthropometric ratios: The newborn body composition study of the INTERGROWTH-21st project. Pediatr. Res..

[39] Pereira-da-Silva L., Abecasis F., Virella D., Videira-Amaral J.M. (2009). Upper Arm Anthropometry Is Not a Valid Predictor of Regional Body Composition in Preterm Infants. Neonatology.

[40] Daly-Wolfe K.M., Jordan K.C., Slater H., Beachy J.C., Moyer-Mileur L.J. (2015). Mid-Arm Circumference Is a Reliable Method to Estimate Adiposity in Preterm and Term Infants. Pediatr. Res..

[41] McNelis K., Fu T.T., Poindexter B. (2017). Nutrition for the extremely preterm infant. Clin. Perinatol..

[42] Alja’nini Z., Merlino-Barr S., Brumfiel A., McNelis K., Viswanathan S., Collin M., Groh-Wargo S. (2021). Effect of parenteral nutrition duration on patterns of growth and body composition in very low-birth-weight premature infants. JPEN J. Parenter. Enteral Nutr..

[43] Liotto N., Amato O., Piemontese P., Menis C., Orsi A., Corti M.G., Colnaghi M., Cecchetti V., Pugni L., Mosca F. (2020). Protein intakes during weaning from parenteral nutrition drive growth gain and body composition in very low birth weight preterm infants. Nutrients.

[44] Uthaya S., Liu X., Babalis D., Doré C.J., Warwick J., Bell J., Thomas L., Ashby D., Durighel G., Ederies A. (2016). Nutritional evaluation and optimisation in neonates: A randomized, double-blind controlled trial of amino acid regimen and intravenous lipid composition in preterm parenteral nutrition. Am. J. Clin. Nutr..

[45] Li Y., Liu X., Modi N., Uthaya S. (2019). Impact of breast milk intake on body composition at term in very preterm babies: Secondary analysis of the Nutritional Evaluation and Optimisation in Neonates randomised controlled trial. Arch. Dis. Child. Fetal Neonatal Ed..

[46] Lach L.E., Chetta K.E., Gregoski M.J., Katikaneni L.D. (2023). Trends in preterm body composition and neurodevelopmental outcomes after discharge. Neonatology.

[47] McNelis K., Liu C., Ehrlich S., Fields C., Fields T., Poindexter B. (2021). Body composition of very low-birth-weight infants fed fortified human milk: A pilot study. JPEN J. Parenter. Enteral Nutr..

[48] Lingwood B.E., Al-Theyab N., Eiby Y.A., Colditz P.B., Donovan T.J. (2020). Body composition in very preterm infants before discharge is associated with macronutrient intake. Br. J. Nutr..

[49] Simon L., Frondas-Chauty A., Senterre T., Flamant C., Darmaun D., Rozé J.C. (2014). Determinants of body composition in preterm infants at the time of hospital discharge. Am. J. Clin. Nutr..

[50] Robinson D.T., Calkins K.L., Chen Y., Cober M.P., Falciglia G.H., Church D.D., Mey J., McKeever L., Sentongo T. (2023). Guidelines for parenteral nutrition in preterm infants: The American Society for Parenteral and Enteral Nutrition. JPEN J. Parenter. Enteral Nutr..

[51] Raiten D.J., Steiber A.L., Papoutsakis C., Rozga M., Handu D., Proaño G.V., Moloney L., Bremer A.A. (2023). The “Breastmilk Ecology: Genesis of Infant Nutrition (BEGIN)” Project—Executive summary. Am. J. Clin. Nutr..

[52] Groer M.W. (2023). Editorial on human milk as a biological system. J. Hum. Lact..

[53] Ong M.L., Cherkerzian S., Bell K.A., Berger P.K., Furst A., Sejane K., Bode L., Belfort M.B. (2024). Human milk oligosaccharides, growth, and body composition in very preterm infants. Nutrients.

[54] Salas A.A., Gunawan E., Nguyen K., Reeves A., Argent V., Finck A., Carlo W.A. (2023). Early human milk fortification in infants born extremely preterm: A randomized trial. Pediatrics.

[55] Belfort M.B. (2023). Sooner is better: Early human milk fortification for hospitalized preterm infants < 29 weeks. Pediatrics.

[56] Parat S., Raza P., Kamleh M., Super D., Groh-Wargo S. (2020). Targeted breast milk fortification for very low birth weight (VLBW) infants: Nutritional intake, growth outcome and body composition. Nutrients.

[57] McLeod G., Sherriff J., Nathan E., Geddes D., Simmer K. (2016). Comparing different methods of human breast milk fortification using measured v. assumed macronutrient composition to target reference growth: A randomised controlled trial. Br. J. Nutr..

[58] Olhager E., Danielsson I., Sauklyte U., Törnqvist C. (2021). Different feeding regimens were not associated with variation in body composition in preterm infants. J. Matern. Fetal Neonatal Med..

[59] Piemontese P., Liotto N., Mallardi D., Roggero P., Puricelli V., Giannì M.L., Morniroli D., Tabasso C., Perrone M., Menis C. (2018). The effect of human milk on modulating the quality of growth in preterm infants. Front. Pediatr..

[60] Picaud J.C., Decullier E., Plan O., Pidoux O., Bin-Dorel S., Egroo L.D., Chapuis F., Claris O. (2008). Growth and bone mineralization in preterm infants fed preterm formula or standard term formula after discharge. J. Pediatr..

[61] Koo W.W., Hockman E.M. (2006). Posthospital discharge feeding for preterm infants: Effects of standard compared with enriched milk formula on growth, bone mass, and body composition. Am. J. Clin. Nutr..

[62] Amesz E.M., Schaafsma A., Cranendonk A., Lafeber H.N. (2010). Optimal growth and lower fat mass in preterm infants fed a protein-enriched postdischarge formula. J. Pediatr. Gastroenterol. Nutr..

[63] Roggero P., Giannì M.L., Amato O., Liotto N., Morlacchi L., Orsi A., Piemontese R., Taroni F., Morniroli D., Bracco B. (2012). Growth and fat-free mass gain in preterm infants after discharge: A randomized controlled trial. Pediatrics.

[64] Brunton J.A., Saigal S., Atkinson S.A. (1998). Growth and body composition in infants with bronchopulmonary dysplasia up to 3 months corrected age: A randomized trial of a high-energy nutrient-enriched formula fed after hospital discharge. J. Pediatr..

[65] Cooke R.J., Griffin I.J., McCormick K., Embleton N., Faulkner K., Wells J.C., Rawlings D.C. (1999). Feeding preterm infants after hospital discharge: Effect of diet on body composition. Pediatr. Res..

[66] Lee J.J., Beretvas S.N., Freeland-Graves J.H. (2014). Abdominal Adiposity Distribution in Diabetic/Prediabetic and Nondiabetic Populations: A Meta-Analysis. J. Obes..

[67] Rabinowich A., Avisdris N., Yehuda B., Vanetik S., Khawaja J., Graziani T., Neeman B., Wexler Y., Specktor-Fadida B., Herzlich J. (2024). Fetal body composition reference charts and sexual dimorphism using magnetic resonance imaging. Am. J. Clin. Nutr..

[68] Murphy-Alford A.J., Johnson W., Nyati L.H., Santos I.S., Hills A.P., Ariff S., Wickramasinghe V.P., Kuriyan R., Lucas M.N., Costa C.S. (2023). Body composition reference charts for infants from birth to 24 months: Multicenter Infant Body Composition Reference Study. Am. J. Clin. Nutr..

[69] Rodríguez G., Samper M.P., Ventura P., Moreno L.A., Olivares J.L., Pérez-González J.M. (2004). Gender differences in newborn subcutaneous fat distribution. Eur. J. Pediatr..

[70] Uthaya S., Thomas E.L., Hamilton G., Doré C.J., Bell J., Modi N. (2005). Altered adiposity after extremely preterm birth. Pediatr. Res..

[71] Mol N., Zasada M., Kwinta P. (2019). Does type of feeding affect body composition in very low birth weight infants?—A prospective cohort study. Pediatr. Neonatol..

[72] Simon L., Borrego P., Darmaun D., Legrand A., Rozé J.C., Chauty-Frondas A. (2013). Effect of sex and gestational age on neonatal body composition. Br. J. Nutr..

[73] Alur P., Ramarao S. (2022). Sex differences in preterm nutrition and growth: The evidence from human milk associated studies. J. Perinatol..

[74] Ingolfsland E.C., Haapala J.L., Buckley L.A., Demarath E.W., Guiang S.F., Ramel S.E. (2019). Late Growth and Changes in Body Composition Influence Odds of Developing Retinopathy of Prematurity among Preterm Infants. Nutrients.

[75] Scheurer J.M., Zhang L., Plummer E.A., Hultgren S.A., Demerath E.W., Ramel S.E. (2018). Body Composition Changes from Infancy to 4 Years and Associations with Early Childhood Cognition in Preterm and Full-Term Children. Neonatology.

[76] Jussinniemi L., Kulmala M.K., Aakvik K.A.D., Benum S.D., Jørgensen A.P.P., Balasuriya C.N.D., Stunes A.K., Syversen U., Indredavik M.S., Andersson S. (2024). Body composition in adults born preterm with very low birth weight. Pediatr. Res..

[77] Matinolli H.M., Hovi P., Männistö S., Sipola-Leppänen M., Eriksson J.G., Mäkitie O., Järvenpää A.L., Andersson S., Kajantie E. (2015). Early Protein Intake Is Associated with Body Composition and Resting Energy Expenditure in Young Adults Born with Very Low Birth Weight. J. Nutr..

[78] McClave S.A., Martindale R.G. (2024). Browning of white adipose tissue may be an appropriate adaptive response to critical illness. JPEN J. Parenter. Enteral Nutr..

[79] Algotar A., Shaikhkhalil A.K., Siler-Wurst K., Sitaram S., Gulati I., Jadcherla S.R. (2018). Unique Patterns of Body Composition and Anthropometric Measurements During Maturation in Neonatal Intensive Care Unit Neonates: Opportunities for Modifying Nutritional Therapy and Influencing Clinical Outcomes. JPEN J. Parenter. Enteral Nutr..

[80] Lücke L., Fusch C., Knab K., Schäfer S., Zimmermann J.L., Müser U.F., Meis A., Weiß S.L., Fusch A.S., Rochow N. (2024). Reproducibility of Air Displacement Plethysmography in Term and Preterm Infants-a Study to Enhance Body Composition Analysis in Clinical Routine. Nutrients.

[81] van de Lagemaat M., Ruys C.A., Muts J., Finken M.J., Rotteveel J., Goudoever J.B., Lafeber H.N., Akker C.H., Levie N.S., Boonstra V. (2024). Growth and Body Composition of Infants Born Moderate-to-Late Preterm Fed a Protein- And Mineral-Enriched Postdischarge Formula Compared with a Standard Term Formula Until 6 Months Corrected Age, a Randomized Controlled Trial. Am. J. Clin. Nutr..

[82] Perrin T., Pradat P., Larcade J., Imbert M.M., Diez B.P., Picaud J.C. (2023). Postnatal Growth and Body Composition in Extremely Low Birth Weight Infants Fed with Individually Adjusted Fortified Human Milk: A Cohort Study. Eur. J. Pediatr..

[83] Salas A.A., Jerome M., Finck A., Razzaghy J., Laney P.C., Carlo W.A. (2022). Body Composition of Extremely Preterm Infants Fed Protein-Enriched, Fortified Milk: A Randomized Trial. Pediatr. Res..

[84] Kuehn D., Zeisel S.H., Orenstein D.F., German J.B., Field C.J., Teerdhala S., Knezevic A., Patil S., Donovan S.M., Lönnerdal B. (2022). Effects of a Novel High-Quality Protein Infant Formula on Energetic Efficiency and Tolerance: A Randomized Trial. J. Pediatr. Gastroenterol. Nutr..

[85] Maas C., Mathes M., Bleeker C., Vek J., Bernhard W., Wiechers C., Peter A., Poets C.F., Franz A.R. (2017). Effect of increased enteral protein intake on growth in human milk-fed preterm infants: A randomized clinical trial. JAMA Pediatr..

[86] Kawai M. (2018). Reevaluation of protein intake for preterm infants. Am. J. Perinatol..

[87] Toftlund L.H., Halken S., Agertoft L., Zachariassen G. (2018). Early nutrition and signs of metabolic syndrome at 6 years of age in children born very preterm. Am. J. Clin. Nutr..

[88] Alja’nini Z., McNelis K.M., Viswanathan S., Goddard G.R., Merlino-Barr S., Collin M., Groh-Wargo S. (2021). Infant body composition assessment in the neonatal intensive care unit (NICU) using air displacement plethysmography: Strategies for implementation into clinical workflow. Clin. Nutr. ESPEN.

[89] Lücke L.A., Rochow N., Knab K., Schäfer S., Zimmermann J.L., Meis A., Weiß S.L., Fusch A.S., Müser U.F., Fusch C. (2024). Body Composition Analysis of the Clinical Routine Using Air Displacement Plethysmography: Age-Group-Specific Feasibility Analysis Among Preterm Infants. Nutrients.

[90] Bell K.A., Ramel S.E., Robinson D.T., Wagner C.L., Scottoline B., Belfort M.B. (2022). Body Composition Measurement for the Preterm Neonate: Using a Clinical Utility Framework to Translate Research Tools Into Clinical Care. J. Perinatol..

[91] Brennan A.M., Murphy B.P., Kiely M.E. (2016). Optimising Preterm Nutrition: Present and Future. Proc. Nutr. Soc..

